# An Aquaphotomics Approach for Investigation of Water-Stress-Induced Changes in Maize Plants

**DOI:** 10.3390/s23249678

**Published:** 2023-12-07

**Authors:** Daniela Moyankova, Petya Stoykova, Petya Veleva, Nikolai K. Christov, Antoniya Petrova, Stefka Atanassova

**Affiliations:** 1 AgroBioInstitute, Agricultural Academy, 1164 Sofia, Bulgaria; dmoyankova@abi.bg (D.M.); pstoykova@abi.bg (P.S.); nikolai_christov@abi.bg (N.K.C.); 2 Faculty of Agriculture, Trakia University, 6000 Stara Zagora, Bulgaria; petya.veleva@trakia-uni.bg (P.V.); antoniya.petrova@trakia-uni.bg (A.P.)

**Keywords:** maize plant, water stress, NIR spectra, aquagrams

## Abstract

The productivity of plants is considerably affected by various environmental stresses. Exploring the specific pattern of the near-infrared spectral data acquired non-destructively from plants subjected to stress can contribute to a better understanding of biophysical and biochemical processes in plants. Experiments for investigating NIR spectra of maize plants subjected to water stress were conducted. Two maize lines were used: US corn-belt inbred line B37 and mutant inbred XM 87-136, characterized by very high drought tolerance. After reaching the 4-leaf stage, 10 plants from each line were subjected to water stress, and 10 plants were used as control, kept under a regular water regime. The drought lasted until day 17 and then the plants were recovered by watering for 4 days. A MicroNIR OnSite-W Spectrometer (VIAVI Solutions Inc., Chandler, AZ, USA) was used for in vivo measurement of each maize leaf spectra. PLS models for determining drought days were created and aquagrams were calculated separately for the plants’ second, third, and fourth leaves. Differences in absorption spectra were observed between control, stressed, and recovered maize plants, as well as between different measurement days of stressed plants. Aquagrams were used to visualize the water spectral pattern in maize leaves and how it changes along the drought process.

## 1. Introduction

The influence of environmental stress factors on plant physiology has been the subject of rigorous research over the years and a significant number of studies have been reported [1,2]. Some of the mechanisms used by plants to respond and try to adapt and recover their homeostasis have also been described [3,4]. In general, plants have developed smart and sophisticated mechanisms to overcome stress [5], although these properties are more or less pronounced depending on the plant species [6]. Plants respond by changes in metabolism, gene expression, and adjustment of developmental processes. The common theme among different types of stress conditions is the generation of reactive oxygen species in cells. These particles act as aggressive disruptors on the most important cellular structures (e.g., membrane systems) and nucleic acids [7,8,9,10]. To alleviate the negative effect of the radicals’ plants, they trigger enzymatic antioxidative systems (e.g., superoxide dismutases, peroxidases, catalases, and phenol oxidase). Another route to withstand the severe destructive influence of the generated radicals and alleviate the overall negative impact is the involvement of compounds with strong reductive potential (phenols, polyamines, flavonoids, glutathione, etc.).

Maize (*Zea mays* L.) is among the most exploited cereal crops with great importance all around the world. Despite that, its production is limited by drought [11,12], which can bring a decrease of 25–30% in yield in some arid zones [13]; it is widely cultivated for direct use as food and feed, as well as for processed production of industrial compounds. When drought stress is in place, the maize yield losses vary from 30 to 90% depending on (1) the stage of the plant as well as (2) the intensity and prolongation of the drought period. As a result, the flowering and grain-filling stages are also severely affected [14]. Maize is frequently cultivated without irrigation in dry areas, with 300–500 mm of annual rainfall despite the fact that this level is below the critical limit necessary to reach a good crop yield [14]. Maize plants are sensitive to drought stress during all developmental stages from seed germination and seedling emergence through vegetative growth and reproductive phases. Therefore, improvement in drought tolerance is a major focus of research for maize breeding [15]. It was shown that applying a solution with 0.99 MPa of osmotic potential to maize seeds significantly affects the progress of germination and post-germination phases of the treated plants [16]. The most drought-sensitive period in maize development is between emergence (VE) and the 5-leaf (V5) stage seedling [17]. Environmental stress occurring during the seedling stage can result in crop failure [18,19]. Although significant improvement of drought tolerance during the reproductive stage of maize plants was achieved recently by application of various omics approaches [20], little attention is paid to the improvement of drought tolerance at the seedling stage due to its very complex nature. Therefore, a profound understanding of the physiological mechanisms behind vegetative drought tolerance at the seedling stage will be of high value.

Plant stress detection is very important to increase crop yield. Some of the methods for quantitative estimation of stress effects, such as metabolomics methods, provide very accurate data, but they are sampling destructive and very slow, which prevents dynamical studies.

Near-infrared (NIR) spectroscopy is a non-destructive rapid method for qualitative and quantitative analyses in different scientific fields—in the industry, medicine, agriculture, etc. NIR spectroscopy is less expensive than conventional methods because it does not use special consumables and chemicals after instrument calibration, requires little or no sample preparation, and can be used for online measurements. Data on the concentration of several components can be obtained with only one measurement. Another advantage of the method is that the analyzed samples can contain high water contents, and therefore this method has the possibility for in vivo application in living systems.

Recently, spectroscopy in the visible and near-infrared region was used to investigate the effect of abiotic stress on the base of spectral reflectance [21,22,23,24]. One of the used approaches is the development of vegetation indices, typically used in remote sensing. Another approach is to use chemometric methods to extract information from the near-infrared spectra of plants and food for the determination of quantitative parameters and for classification [25,26,27,28].

Aquaphotomics is a novel scientific approach based on near-infrared spectra of water in different kinds of materials, mainly biological samples with high water content, and analysis of changes in water spectra caused by the influence of different factors [29,30,31]. Aquaphotomics analysis of water–light interactions is a new source of information for a better understanding of the biological world. The combination of NIRS and aquaphotomics has already been used for the non-destructive investigation of aqueous and biological systems, for the evaluation of fresh and processed fruits and vegetables quality [32,33,34,35,36,37,38], plant abiotic stress and diseases [39,40,41,42].

In this paper, we use a non-destructive, portable NIR spectrometer to analyze the response to drought stress of two maize inbred lines—a US corn-belt B37 and a mutant XM 87-136 with very high drought tolerance by an aquaphotomics approach.

## 2. Materials and Methods

### 2.1. Plant Materials and Experimental Conditions

Two maize (*Zea mays* L.) inbred lines were included in the present study, the well-known US corn-belt inbred line B37 and a mutant inbred, developed at Maize Research Institute, Kneja, Bulgaria. We focused on inbred lines since they are genetically uniform and should give lower plant-to-plant variation in the experiments. The mutant line XM 87-136 was developed by chemical mutagen treatment of B37 dry seeds followed by recurrent reciprocal mutation breeding to fix the desired mutant traits [43,44]. In addition to its high yield and combining the ability to produce high-yielding hybrids, the mutant line XM 87-136 is characterized by very high drought tolerance. XM 87-136 is a parental line of one of the most drought-tolerant maize hybrids bred in Bulgaria that has now been on the market for more than 20 years. The inbred line B37Ht (Co-op ID: 3406-007) was obtained from the Maize Genetics Cooperation Stock Center (http://maizecoop.cropsci.illinois.edu, accessed on 20 October 2023). Both lines used in the present study were maintained in a homozygous state by more than 10 generations of self-fertilization (selfing) at MRI-Kneja.

Seeds were surface sterilized with 5% commercial bleach (Domestos) for 10 min, washed three times with distilled water, and placed on wet filter paper. After germination, seedlings were transferred to a pot (d 10 cm) with soil. Plants were grown well watered at 28/25 °C day/night temperature, 50 ± 5% relative air humidity, and at 150 µmol m^−2^ s^−1^—photon flux density of light intensity for 16 h in a controlled growth chamber (MLR-351, SANYO, Osaka, Japan).

Drought stress was induced on pot plants at the 4-leaf stage by withholding water for 17 days followed by rewatering for 4 days. Measurements were made on days 3, 7, 10, 12, 14, and 17 during drought stress and on days 3 and 4 after rewatering. Control plants were well watered during the treatment. A total of 10 plants from each line were subjected to water stress, and 10 plants were used as controls and kept under a regular water regime.

**Figure 1:**
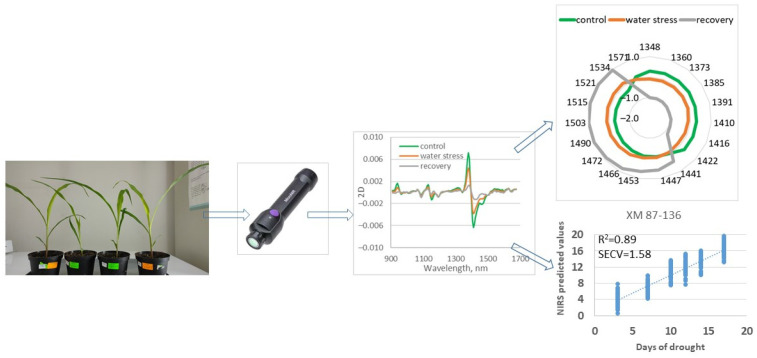
Scheme of experimental design.

The scheme of the experimental design is presented in Figure 1.

### 2.2. Relative Water Content (RWC)

RWC of the 4th leaf at various time points of stress and recovery was calculated using the following formula [45]:

(1) $$RWC\%=\left[\left(FW-DW\right)/\left(FTW-DW\right)\right]\times 100,$$

where FW is the fresh weight of the leaf, FTW is the fresh turgor weight measured after immersion of the leaf in distilled water for 24 h at room temperature and DW is the dry weight measured gravimetrically after drying at 80 °C in an oven for 48 h. Data are shown as the mean± standard deviation (SD) of four biological replicates.

### 2.3. Plant Height

The plant height (cm/plant) was measured as a distance from the soil surface to the upper end of the longest leaf at every time point during drought stress and recovery. Data are shown as the mean ± standard deviation (SD) of ten biological replicates.

### 2.4. NIRS Measurements

A portable, handheld MicroNIR OnSite-W spectrometer (VIAVI Solutions Inc., Chandler, AZ, USA) in the diffuse reflectance mode was used for spectral acquisition in the 908–1670 nm spectral range, with an approximately 7 nm resolution step. The second, third, and fourth leaves from each plant were measured. Each leaf was measured at three different positions. A dark 5 mm thick plate under the measuring maize leaves was used to ensure uniform measurement conditions and to avoid external interference.

### 2.5. Aquaphotomics and Multivariate Data Analysis

Pirouette 4.5 software (Infometrix, Inc., Bothell, WA, USA) was used for spectral data processing. Second derivative preprocessing of spectral data was applied, based on a Savitzky–Golay polynomial filter. The equations for the determination of days of water stress were created by partial least square (PLS) regression and the second derivative transformation of spectral data. Cross-validation was used for the determination of an optimum number of PLS factors in the models—the number of factors that corresponded to the lowest standard error of cross-validation SECV. Three leave-out samples were used in the cross-validation procedure because three replicates of the same leaf were measured.

Aquagram is a radar chart with coordinates, connected with specific water absorption bands of free water, dimers, trimers, solvation shells, etc., named water matrix coordinates (WAMACs) [29]. To calculate the aquagrams coefficients, first spectral data were transformed by multiplicative scatter correction (MSC) to reduce scattering effects due to the different thickness and surface properties of leaves. After that, normalized absorbance values at several wavelengths, Aq were calculated using the equation:

(2) $${Aq}_{\lambda }=\frac{A_{\lambda }-\mu_{\lambda }}{\sigma_{\lambda }}$$

where $A_{\lambda }$ is the absorbance at wavelength λ, $\mu_{\lambda }$ is the mean value, and $\sigma_{\lambda }$ is the standard deviation of all spectra at wavelength λ, respectively.

Aquagrams were calculated using the new 19 WAMACs, proposed by Vitalis et al. [46].

### 2.6. Statistical Data Analysis

Statistical data analysis included multivariate ANOVA to calculate significant differences among aquagram values at various WAMACS. Significance was determined using post hoc multiple comparisons with either Dunnett’s T3 or Tukey tests, depending on the results of Levene’s test for equality of error variances, at *p*-values of ≤0.05, 0.01, and 0.001. IBM SPSS Statistics 26.0 was used to process the data.

## 3. Results and Discussion

### 3.1. Physiological Changes during Drought

**Figure 2:**
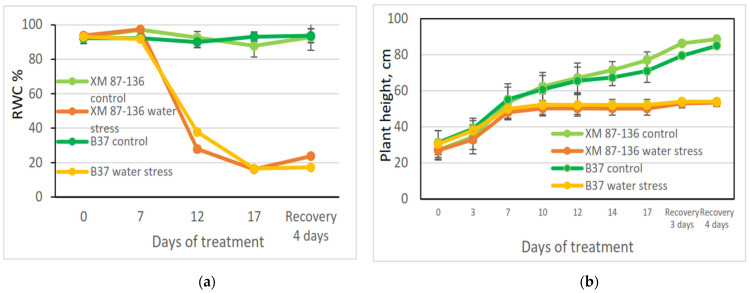
Changes in leaf RWC (**a**) and plant growth (**b**) of inbred maize B37 and XM 87-136 during drought stress and recovery. Data are presented as the mean values ± standard deviation. (**a**) Relative water content (RWC) of tested maize lines; (**b**) plant growth of tested maize lines.

**Figure 3:**
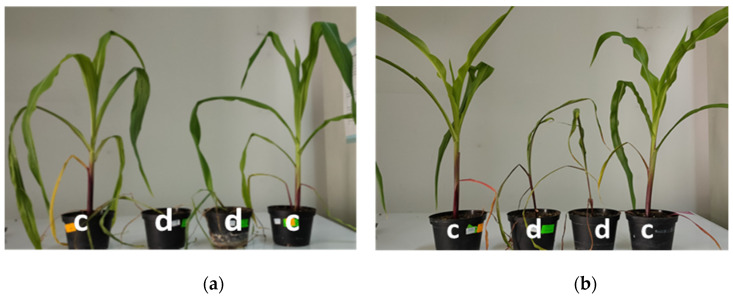
Phenotype response of inbred maize line B37 and XM 87-136 on the 17th day of drought stress treatment. c—control plants; d—maize plants on the 17th day of drought stress. (**a**) B37—day 17; (**b**) XM 87-136—day 17.

Two inbred maize lines—B37 (non-tolerant) and XM 87-136 (tolerant)—were subjected to water stress. Control plants from both lines have approximately 92% relative water content (RWC) (Figure 2a). When plants were exposed to drought, RWC for both lines decreased after the 7th day and reached 16% on the 17th day (Figure 3) and the leaves were not able to recover on the 4th day after rewatering. Slightly better was the recovery for the line XM 87-136.

Maize growth during drought showed the same pattern for both analyzed genotypes—plants stopped growing after the 7th day of stress treatment compared to controls (Figure 2b).

### 3.2. NIR Spectra of Maize Leaves in the Process of Water Stress

**Figure 4:**
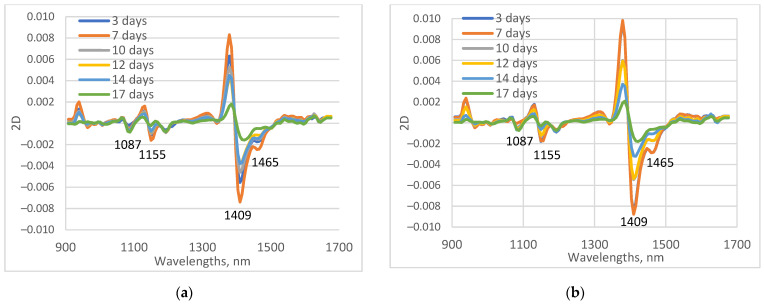
Average second derivative spectra (2D) of leaves of B37 and XM 87-136 maize lines in process of water stress. (**a**) B37; (**b**) XM 87-136.

Average second derivative of maize leaf spectra, measured from 3 to 17 days of drought, are shown in Figure 4 for both investigated maize lines. Absorption patterns of the two maize inbred lines were very similar. The spectra were dominated by the absorption band of water in the first overtone region between 1400 and 1500 nm. The biggest differences in the spectra for both tested maize lines could be observed at 1409 nm, connected with the absorbance of free water molecules. The absorption decreases with increasing the period of water stress. Absorption at approximately 1155 nm is connected with water absorption—a combination of the first overtone of O-H stretching and O-H bending vibration [37]. Absorption of maize leaves at 1087 nm also shows a dependence on drought days.

El-Hendawy et al. [47] also found a high positive correlation in the region 1392–1550 nm between the spectral reflectance of spring wheat, subjected to different irrigation rates and leaf water potential, and a high negative correlation with relative water content, and equivalent water thickness. In the experiment for non-destructive detection of water stress and estimation of relative water content in maize [48], it was found that the coefficient of variation in the reflectance spectra of water-stressed plants had a pronounced peak at approximately 1450 nm. The reflectance of control plant leaves was virtually invariant. Das et al. [49], in an experiment with 10 different rice genotypes, investigated water absorption bands, indices, and multivariate models for the development of non-destructive water-deficit stress phenotyping protocols using VNIR spectroscopy. Among the water absorption features at 970, 1200, 1400, and 1900 nm, 1400 nm was found to be the best for the estimation of leaf water content.

The main visible difference between the two maize lines was the magnitude of absorption at 1409 nm. For all days, the absorbance values for the drought-tolerant mutant line were higher than those for B37.

**Figure 5:**
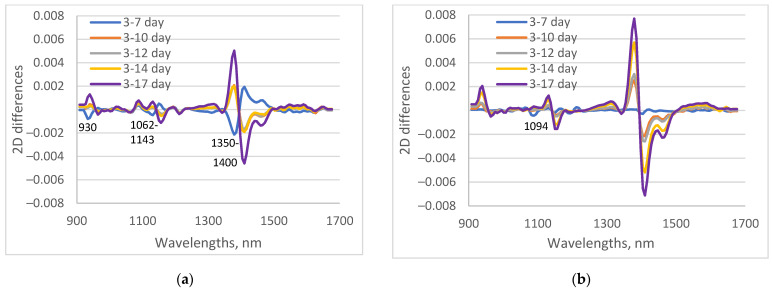
Differences between second derivative spectra of water-stressed leaves, measured at day 3, and measured at 7, 10, 12, 14, and 17 days. (**a**) B37, 3rd leaf; (**b**) XM 87-136, 3rd leaf.

The differences between spectra of maize lines, measured at 3 days of water stress and other days were calculated and presented in Figure 5. The stress responses of the two investigated maize lines now varied. For the drought-tolerant line, there are almost no differences in the spectrum until day 7 of drought. In the case of the B37, there are differences between the 7th and the 3rd day of water stress, especially at 930 nm, and the region 1350–1400 nm. The absorption at approximately 930 nm suggesting bond with either the C-H or O-H group. According to Rajkumar et al. [50], absorption at 930–935 nm is connected with O-H stretch (water solvation shell). The changes in the absorption in the area 1350–1400 nm are connected with weakly hydrogen-bonded water and trapped water or water vapor. This may indicate a loss of moisture through transpiration or a different content of trapped water, which is more significant for B37 plants [38,46]. Therefore, B37 plants undergo some physiological changes at the beginning of water stress that are different from those of XM 87–135 plants. These changes have not yet affected the physiological data at day 7 of drought, but have altered the spectral characteristics measured for B37 plants. Additionally, differences were observed in the area from 1062 to 1143 nm, mainly due to O-H and C-H vibrations [51]. This showed some differences in the processes of water stress between the two maize inbreds.

### 3.3. NIR Spectra of Control, Water-Stressed, and Recovery Maize Leaves

**Figure 6:**
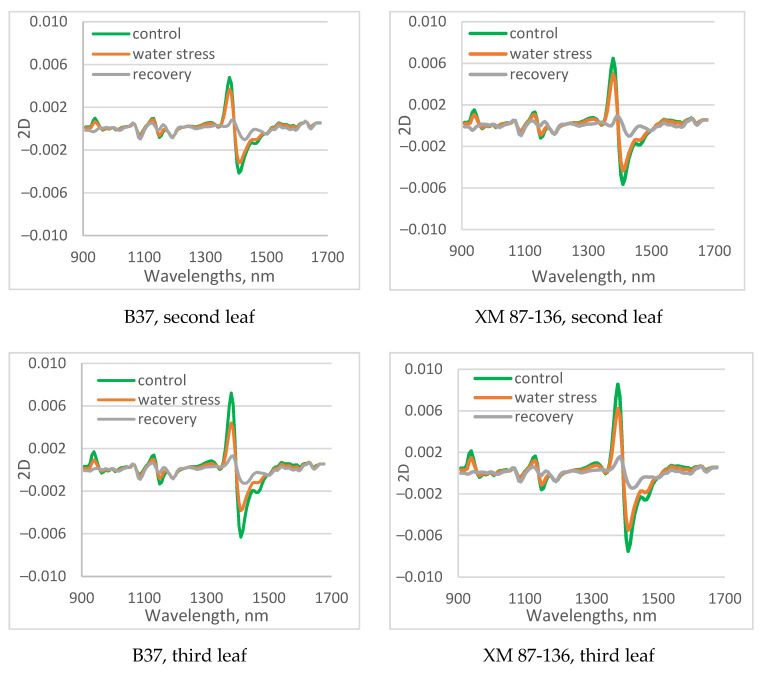

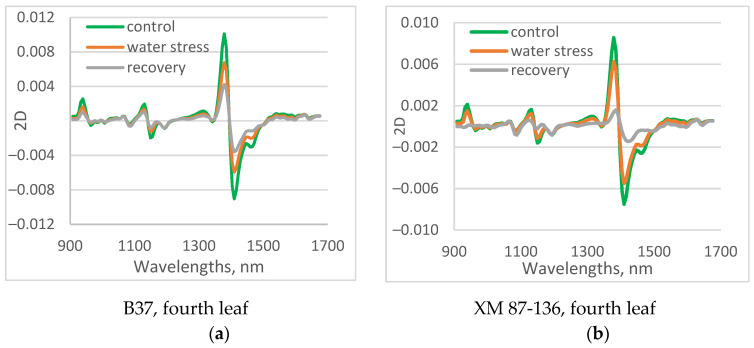
Average second derivative spectra of control, water-stressed, and recovery second, and fourth leaves of B37 (**a**) and XM 87-136 (**b**) maize lines.

The second derivatives of average maize leaf spectra of control, water-stressed, and recovery plants are presented in Figure 6. There were clear differences among spectra of control, water-stressed, and recovered maize leaves for both investigated inbred lines.

The most intensive absorption was found in the first overtone region of water between 1400 and 1500 nm. Again, the absorbance intensity at 1409 nm and 1155 nm was greater in the control plants from drought-tolerant inbred XM 87-136, compared to the B37 line. The same finding was observed for the magnitude of absorption at 1409 nm of water-stressed plants. The spectra of plants recovered by watering for 4 days are very similar to those of water-stressed plants on the 17th day. This indicates that plants do not fully recover after 17 days of drought.

There were differences in the spectra of the second, third, and fourth leaves, mainly related to the magnitude of absorption.

Early detection of water stress is essential for efficient crop management. The obtained results confirm the possibility of using crop spectral reflectance in the visible and near-infrared regions to assess water stress [52,53,54,55]. The results of the analysis of spectral characteristics of control and water-stressed maize plants showed detectable differences after 3 days of drought for conventional and 7 days for drought-tolerant lines. In addition to monitoring, these results could also be used in breeding programs for fast and non-destructive selection of drought-tolerant maize varieties. 

### 3.4. Aquaphotomics Analysis

**Figure 7:**
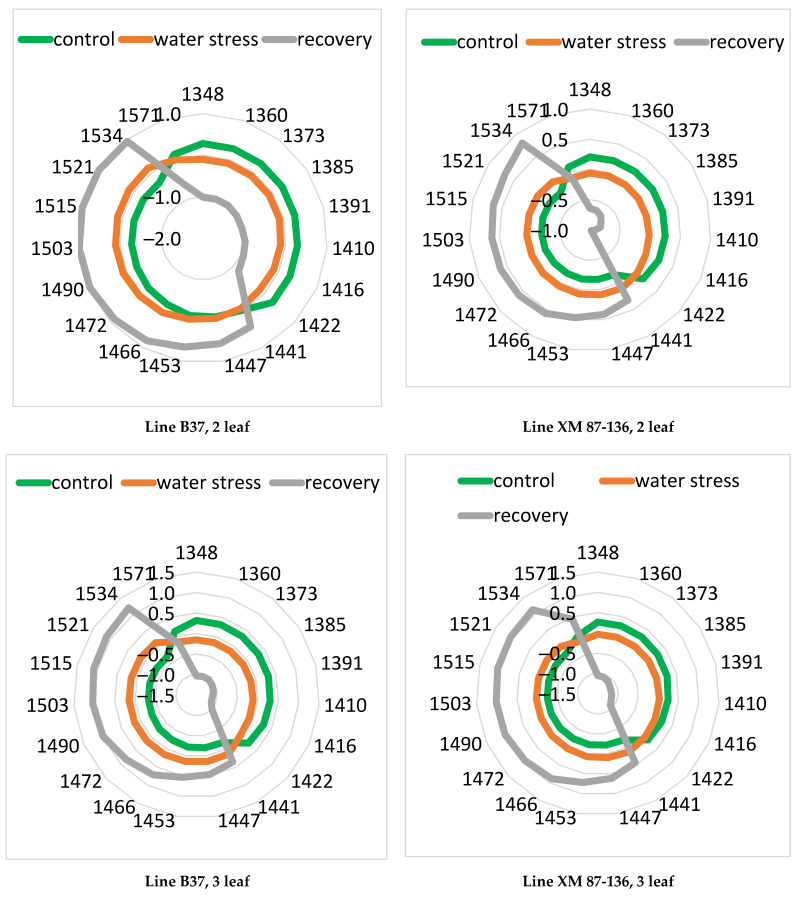

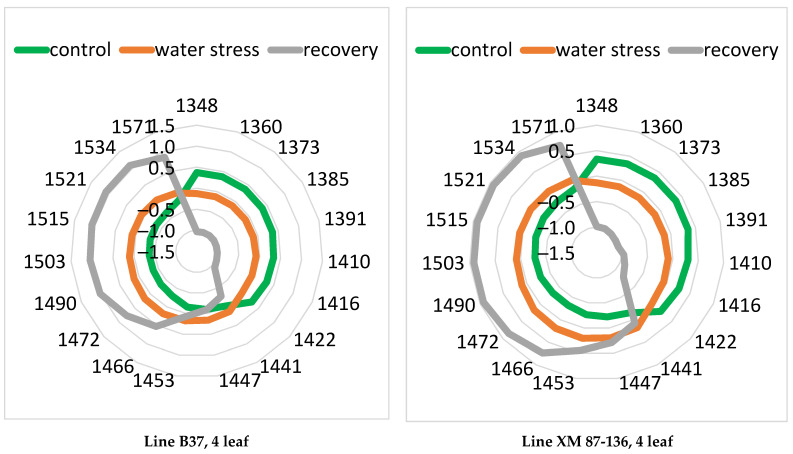
Aquagrams of the different leaves of inbred maize lines—B37 and XM 87-136—during water stress, recovery, and control conditions.

Aquagrams were calculated to further investigate the changes in maize leaves caused by water stress. We used new 19 WAMACs, proposed by Vitalis et al. [44]. There is consistency in the shape and features of the aquagrams for the two maize cultivars and leaves studied. The comparison of water spectral patterns of control, water-stressed, and recovery plants provided clear differences for both maize line leaves (Figure 7).

In all cases, the values for the control plants in the range 1360–1422 nm were greater than those for the water-stressed plants. WAMACS in this range—1360 nm (C2), 1373 nm (C3), and 1385 nm (C4), are related to proton hydration, ion hydration, and trapped water. Absorption at 1409 nm is connected with free water. This indicated the loss of water in water-stressed leaves. Between 1441 and 1533 nm, we observed an inverse relationship—the values for the water-stressed plants are greater than those of the control plants. The water absorbance in this region is indicative of strongly molecularly bound water (molecules with two hydrogen bonds at 1466 nm, molecules with three hydrogen bonds at 1478 nm, and molecules with four hydrogen bonds at 1490 nm). Other WAMACs in this region were connected with the interaction of protein and water at 1466 nm, and water and cellulose at 1503, 1521, and 1534 nm [46,56]. Aquagrams of recovery leaves were very different from those of control or water-stressed plants and indicated the loss of liquid water and strongly bound crystalline water at wavelengths above 1500 nm. The remaining water in these leaves is bound mainly to the cellulose components in the leaves.

Significant differences among aquagram values at various WAMACs for the third maize leaf in both cultivars can be observed in Table 1. Significant differences between control and water-stressed plants of the line XM 87-136 with different statistical strengths were found for most of the WAMACs except at 1416, 1422, and 1571 nm. Statistical significance was at level p in the range 0.001 ÷ 0.01 at 1348, 1360, 1373, 1441, 1447, and 1443 nm, and for the rest of WAMACs at level p in the range 0.01 ÷ 0.05. Statistical significant differences of greatest strength between control and recovery plants were found for most of the WAMACs. Regarding the B37 line, significant differences between control and water-stressed plants were obtained for all the WAMACs. The strongest statistical significance for the WAMACs was in the range 1348–1410 nm and 1466–1534 nm. Significant differences of greatest strength between control and recovery plants were found for all wavelengths except 1571 nm, and between water-stressed and recovery plants except 1441, 1447, and 1571 nm. Similar results were obtained for the second and fourth leaves.

The results of the statistical significance analysis between the values of the aquagram coefficients again confirm the existence of differences in the water stress processes between the two studied maize inbred lines. The statistical significance between the coefficients of the aquagrams for the drought-resistant line XM 87-136 is lower than that for the B37.

**Figure 8:**
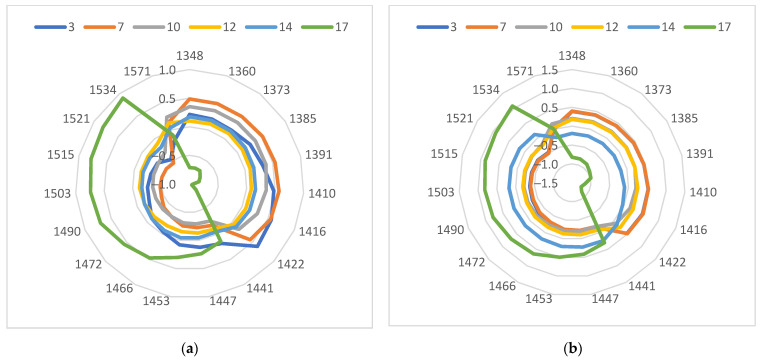
Aquagrams for water-stressed inbred maize lines—B37 and XM 87-137—at different days of water deprivation. (**a**) B37; (**b**) XM 87-136.

For further investigation of changes in leaves during water stress, aquagrams were calculated for different drought days (Figure 8). There is a smooth decrease in the values of the aquagram coefficients in the range of 1347–1442 nm for XM 87-136 water-stressed plants. The values for days 3 and 7 are very close, as are those for days 10 and 12. The values for days 3 to 10 in the 1440–1533 nm range are quite close. Very different from the others is the aquagram obtained for the leaves of the plants on day 17 of the water stress.

The character of the aquagrams for days 3 and 7 for B37 plants was different compared to those for the line XM 87-136. This confirms the observed differences in early drought processes between the two maize lines. They are especially significant in the range 1409–1453 nm. The water absorption at 1441 and 1447 nm is important in desiccation processes and abiotic stress [35,42].

### 3.5. PLS models for Determination of Days of Drought

**Figure 9:**
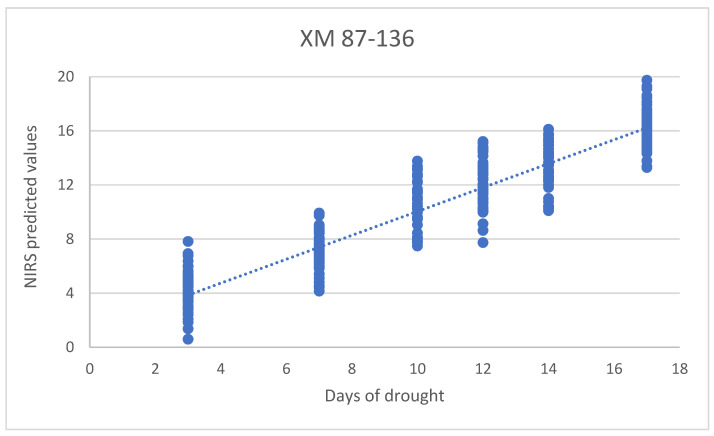
PLSR model for determination of days of drought of XM 87-136 plants.

The PLS models for the determination of days of drought were calculated separately for the data acquired for lines B37 and XM 87-136, using spectral regions from 1300 to 1600 nm, and their performances were presented in Table 2 and Figure 9. The predictions of the days of drought were successful with slightly less accuracy for the B37 inbred—SECV = 1.77 compared to SECV = 1.58 for the XM 87-136 line.

**Figure 10:**
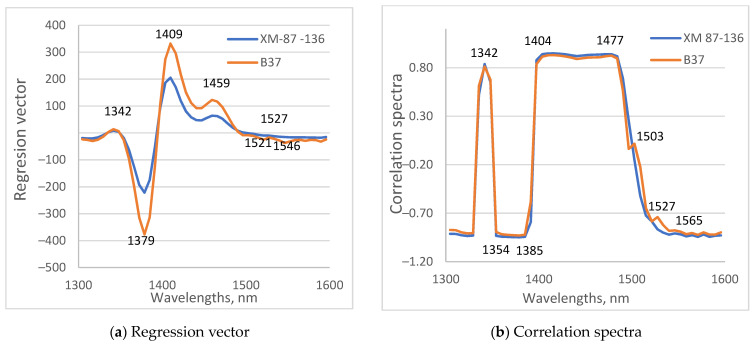
Regression vector (**a**) and correlation spectra (**b**) of PLS models for determination of days of drought.

Regression vectors and correlation spectra from PLS procedures were investigated to find important wavelengths for the determination of days of drought. A regression vector plot is a line plot of coefficients in the model versus wavelengths. The correlation spectrum shows a correlation between the determined parameter and spectral information. Both models for B37 and XM 87-136 provided similar regression vectors and correlation spectra (Figure 10). The most influential variables were at 1342, 1379, 1409 and approximately 930 1459 nm, which are close to WAMAC coordinates C1, C4, C5, and C9, respectively. In the area 1350–1500 nm, there was only a difference in the magnitude of regression vector coefficients. They were bigger for B37 compared to those for the XM 87-136 line. This is related to the difference in absorption values for the two types of maize plants. Small differences existed at 1521, 1527, and 1546 nm. The differences in correlation spectra were most significant in the region 1500–1600 nm, similar to the result of regression vectors. They appear at 1503 and 1527 nm in the correlation spectra of the model for the B37 line. Absorption at 1503 nm can be associated with strongly bound water. In previous investigations absorption at 1527 nm is connected with structural–water and water–cellulose interactions [57].

The results of the analysis of the regression vectors and the correlation spectra confirm the existence of differences in the spectral characteristics of the leaves of the two studied maize inbred lines. The differences are related to changes in water content and water structure during drying.

## 4. Conclusions

This study was conducted to investigate the changes during water stress of maize plants using an aquaphotomic approach. Spectra of plants from two inbred lines, one of them drought resistant, were analyzed.

Differences in absorption spectra in the first overtone water region from 1300 and 1600 nm were observed between control, water-stressed, and recovered maize plants, as well as between different days of stressed plants. Aquagrams clearly visualize the spectral difference. The spectral differences showed a loss of free and weakly bound water in the process of water stress. The remaining water is in the bound state with a high number of hydrogen bonds or bonded to the structural elements of plants. This statement is also confirmed by the analysis of the results of the PLS models for determining the days of drought. Aquagrams and spectra of two investigated maize lines also show the differences between the two inbred lines connected with drought tolerance.

The results of this pilot study demonstrated the potential of the aquaphotomics approach to better understand the process of water stress in plants. This can also be used as an additional approach in breeding programs for the selection of drought-tolerant maize varieties.

## Figures and Tables

**Table 1:** Multivariate ANOVA results for aquagrams for the third leaf of the XM 87-136 and B37 lines.

XM 87-136				B37			
Wavelength	Control	Water Stress	Recovery	Wavelength	Control	Water Stress	Recovery
1347.899	a	a		1347.899	a	a	
	b		b		b		b
		c	c			c	c
1360.288	a	a		1360.288	a	a	
	b		b		b		b
		c	c			c	c
1372.677	a	a		1372.677	a	a	
	b		b		b		b
		c	c			c	c
1385.065	a	a		1385.065	a	a	
	b		b		b		b
		c	c			c	c
1391.26	a	a		1391.26	a	a	
	b		b		b		b
		c	c			c	c
1409.843	a	a		1409.843	a	a	
	b		b		b		b
		c	c			c	c
1416.037	ns	ns		1416.037	a	a	
	b		b		b		b
		c	c			c	c
1422.231	ns	ns		1422.231	a	a	
	b		b		b		b
		c	c			c	c
1440.814	a	a		1440.814	a	a	
	b		b		b		b
		ns	ns			ns	ns
1447.009	a	a		1447.009	a	a	
	b		b		b		b
		c	c			ns	ns
1453.203	a	a		1453.203	a	a	
	b		b		b		b
		c	c			c	c
1465.592	a	a		1465.592	a	a	
	b		b		b		b
		c	c			c	c
1471.786	a	a		1471.786	a	a	
	b		b		b		b
		c	c			c	c
1490.369	a	a		1490.369	a	a	
	b		b		b		b
		c	c			c	c
1502.758	a	a		1502.758	a	a	
	b		b		b		b
		c	c			c	c
1515.147	a	a		1515.147	a	a	
	b		b		b		b
		c	c			c	c
1521.341	a	a		1521.341	a	a	
	b		b		b		b
		c	c			c	c
1533.73	a	a		1533.73	a	a	
	b		b		b		b
		c	c			c	c
1570.896	ns	ns		1570.896	a	a	
	b		b		ns		ns
		c	c			ns	ns

Same letters in the same row represent significant differences at *p* < 0.05; ns—insignificant differences. Different colours represent the strength of significant differences as follows: <0.001 in green; 0.001 ÷ 0.01 in blue; 0.01 ÷ 0.05 in yellow; non-significant in grey.

**Table 2:** Statistical parameters of PLS models for determination of days of drought.

Line	PLS Factors	SECV	R^2^cv	SEC	R^2^cal
B37	2	1.77	0.922	1.74	0.923
XM 87-136	2	1.58	0.887	1.58	0.889

## Data Availability

Data are contained within the article.
